# Can the Timing of the Origin of Life Be Inferred from Trends in the Growth of Organismal Complexity?

**DOI:** 10.3390/life16010153

**Published:** 2026-01-16

**Authors:** David A. Juckett

**Affiliations:** Department of Pharmacology & Toxicology, Michigan State University, East Lansing, MI 48842, USA; juckett@msu.edu

**Keywords:** origin of life, panspermia, complexity, time, rate of evolution, allometry, information theory, entropy, enthalpy

## Abstract

The origin of life embodies two fundamental questions: how and when did life begin? It is commonly conjectured that life began on Earth around 4 billion years ago. This requires that the complex organization of RNA, DNA, triplet codon, protein, and lipid membrane (RDTPM) architecture was easy to establish between the time the Earth cooled enough for liquid water and the time when early microorganisms appeared. These bracketing events create a narrow window of time to construct a completely operational self-replicating organic system of very high complexity. Another conjecture is that life did not begin on Earth but was seeded from life-bearing space objects (e.g., asteroids, comets, space dust), commonly referred to as panspermia. The second conjecture implies that life formed somewhere else and was part of the solar nebula, originating from an earlier generation star where there was more time available for the development of life. In this paper, the goal is to provide a hypothetical perspective related to the timing for the origin of pre-biotic chemistry and life itself. Using a form of complexity growth, biological features spanning from the present day back to early life on Earth were examined for trends across time. Genome sizes, gene number, protein–protein binding sites, energy for cell construction, mass of individual cells, the rate of cell mass growth, and a molecular complexity measure all yield highly significant regressions of linearly increasing complexity when plotted over the last 4 Gyr (billion years). When extrapolated back in time, intersections with simple complexities associated with each variable yield a mean value of 8.6 Gyr before the present time. This era coincides with the peak of star and planet formation in the universe. This speculative analysis is consistent with the second conjecture for the origin of life. The major assumptions of such an analysis are presented and discussed.

## 1. Introduction

Most of the literature on the origin of life has focused on how it formed rather than when it formed. The primary assumption is that life started on Earth around the time that liquid water appeared, so the focus has been on how it formed. Many ideas have been presented covering various aspects of this problem. In all cases, scientists are working with limited knowledge of the deep past on Earth. Because of this, there are approaches that explore ideas such as the following: life as information [1,2]; environment conditions in which it is likely for life to start [3]; the chemistry that must exist [4,5,6,7]; the likely biochemistries [8,9,10]; the thermodynamics of the transition to life [11,12]; how long would it take during early Earth [13]; and many more.

Because of this broad spectrum of approaches regarding the origin of life, Malaterre et al. [14] attempted to disentangle the various aspects of these questions. They created a three-dimensional conceptual space of the following: “natural spontaneity”—Could the transition from non-living to living mater occur naturally?; “historical adequacy”—Is the development of life compatible with current knowledge of the Earth; and “similarity with life-as-we-know-it”—Is the most likely form of life current life? They maintain that these dimensions put constraints on which hypotheses are more tractable, and which are more speculative. Their approach highlights the diversity of approaches needed to understand the origin of life as well as the difficulty in reaching conclusions. The primary obstacle to all conjectures, hypotheses, and theories is explaining the origin of the intrinsic complexity of living organisms.

As an alternative to life forming on Earth, there have been suggestions that life was seeded (panspermia) from galactic sources. This puts the origin of life’s mystery in the background as something that happened elsewhere. Steele et al. [15] reviewed this possibility and argued that panspermia has many logical components worth considering. Their arguments are basically indirect but by intertwining panspermia to Lamarkian inheritance they provide a possible path for reaching the complexity of life.

The complexity of life is quite remarkable in scope. A partial summary is shown in Table 1. A more abbreviated version is given in The Astrobiology Primer [16]. Table 1a encompasses the complexity that even a bacterium must possess. This is the challenge all origin of life theories must address. In particular, how did all this happen so quickly, driven only by stochastic forces?

Organism complexity is a difficult concept to define. Many have addressed it in various ways, such as defining indexes based on diversity [17], from first principles based on chemistry [18,19], empirically from genomes, genes, cell numbers, chemical networks [20,21,22], in the framework of epigenetics [23], hierarchical transitions [24,25,26,27,28], and from theoretical constructs involving entropy, information theory, network thermodynamics, and complexity spaces [29,30,31,32,33]. In this paper, it is conceded that an all-encompassing definition of biological complexity may be beyond current scientific knowledge. Rather, an approach based on the rate of change in complexity components is proposed and examined with the goal to provide a perspective related to timing for the origin of pre-biotic chemistry and life itself.

This perspective is based on a speculative proposal that the complexity of life grows monotonically and linearly, on average, from construction of the basic building blocks of life to the most elaborate organisms alive today. To construct this perspective, a phenomenological approach is undertaken. The concepts and definitions are presented followed by non-biological examples. Some of these examples are in the Appendix A, Appendix B, Appendix C and Appendix D. Eight features from components of life are examined with respect to complexity growth over time. Seven of these are projected backward in time to early forms representative of that particular feature. Examining data in this context reveals a trend that the building blocks of life and perhaps early life itself could have begun organizing well before the Sun and Earth existed. Predictions can be made from this trend, but true evidence would require moving beyond this phenomenological approach to the creation of hypotheses for testing.

## 2. Concepts for This Analysis

### 2.1. Assumptions

The assumptions that are fundamental to this analysis are provided in three groups. The first two are the basis for the work that follows, while the third is given as a comparative reference for life originating on Earth.

Analysis-enabling assumptions

- Complexity used in this analysis is as defined in Section 2.3.
- An organism alive today is dominated by the same intrinsic complexity that it contained when it first evolved into a new taxon.

Assumptions for this work

- Chemical complexity and biochemical complexity follow the same laws of nature and grow at the same rate.
- Complexity grows linearly from early chemistry to life today.

Assumptions for origin of life occurring on Earth de novo

- Chemical complexity and biochemical complexity follow the same laws of nature but do not grow at the same rate.
- Chemical complexity grows rapidly to reach the first living organism.
- Biochemical complexity grows ten- to twenty-fold slower during evolution, after the appearance of life.

### 2.2. Earliest Life as Prototypes

Discussions surrounding the origin of life often refer to the Last Universal Common Ancestor (LUCA) [34]. This is postulated to be the progenitor of all life on Earth from which bacteria and archaea arose, followed by their merger in some form to create eukaryotes [35]. Preceding LUCA, the First Universal Common Ancestor (FUCA) has been postulated to be a non-cellular entity of ill-defined complexity that eventually led to LUCA [36]. The purpose of proposing FUCA is to account for the many steps involved in moving from chemistry catalyzed by inorganic substrates to chemistry reproducibly catalyzed by biomolecules (proteins and RNA) that are created from memory polymers (RNA, DNA). Intrinsic to the definition of FUCA are the early stages of the complexity that eventually evolved into pockets of biochemical precursors that could maintain functional stability as well as maintain a consistently low entropy enclave [37,38].

It seems reasonable to consider LUCA, and possibly FUCA, as organism prototypes which would have some similarities with human-constructed prototypes. While human-derived prototypes are the product of organized design, unlike living organisms, the times for development can be compared. Human-derived prototypes take longer to construct than subsequent production versions [39]. They are trial-and-error architectures with novel parts and assemblies that must be acquired or one-off created. After successful prototypes appear, replicative production can occur and modifications over time can begin (a form of evolution) without re-inventing the whole device. The modifications should take less effort and time than designing and building the original prototype. By analogy, biological prototypes may not have attained their complexity any faster than they modified that complexity over time (evolution). Since LUCA would need the complexity shown in Table 1a, it is posited that it took much longer than the time available between a habitable Earth and the appearance of either FUCA or LUCA. As shown below, the growth of complexity over the 4 billion years of evolution is approximately the same amount of complexity growth needed to reach LUCA in the first place, under the assumption of linear complexity growth. For a biological prototype to self-assemble on Earth, that process must occur approximately ten times faster than the modifications driven by the complexity changes in evolution.

### 2.3. Defining Complexity

It is accepted as given that a living organism maintains a high level of complexity against forces capable of destroying that complexity. Furthermore, a living organism has the capacity to create a duplicate copy of itself with the same level of complexity. A full description of the complexity of life seems beyond the capabilities of current scientific knowledge. Therefore, for this analysis, the following working definitions are made related to the concept of living organism complexity:

1. Complexity is an intrinsic property of an entity.
2. Every unique entity type has its own complexity signature. Many “nearly identical” instances of an entity can exist with the same complexity (e.g., individuals within an organism species, cells within a multicellular organism).
3. Complexity can be partitioned into a sum of orthogonal components (eigenvectors).
4. Each orthogonal component has a unique profile of features which represents a subset of all the features that comprise the whole entity.
5. Each feature has an associated variable with a value that denotes count, size, intensity, etc.
6. Each feature value has an associated variance across instances of the entity.
7. For two entities to be different, one or more of their respective feature variables must have variances that do not overlap in a significant manner. Such differences must survive across generations.

Drawing on the axioms, theorems, and lemmas of Nehaniv & Rhodes [40], the following are considered important to this work as well.

1. Every component of complexity can be constructed from components of minimal complexity.
2. Complexity can be additive. That is, total complexity is the sum of orthogonal components of complexity. (See Section 4.4 for an example.)
3. Complexity generally grows smoothly but can jump by factors of ~2. Since complexity is hierarchical, the jump may occur at the level of feature values within a hierarchical level or denote a transition from one hierarchical level to another. An example for such a jump is observed in global energy usage, as shown in Appendix A. This phenomenon also appears in the transition to eukaryotic cells for protein–protein binding, as shown in Section 4.5.

While this work postulates that total complexity can be partitioned into orthogonal components, it is accepted that there is insufficient data to make such an analysis. In the future, Principal Component Analysis (PCA) [41] and similar approaches such as Factor Analysis, Latent Factor Analysis, and Latent Trait Analysis [42,43,44] may be applied with success to this decomposition. A hypothetical scenario, using the automobile as an example, is described in Appendix B.

Since complexity cannot be compactly defined in mathematical form, the time derivative for a feature of complexity is defined instead:

(1) $$\frac{dC\left(x_{i}\right)}{dt}\propto \frac{\frac{dx_{i}}{dt}}{x_{i}},$$

where *C*(*x_i_*) is complexity of feature, *i*, with value, *x*. Equation (1) gives the rate of change in *x* as being proportional to its current value. Populations typically grow in this manner, with the next generation dependent only on the current generation number in a Markov chain fashion. Similarly, evolution is basically the addition of new capabilities on top of what already exists. The variable, *x*, is something quantifiable, countable, measurable, etc.

Equation (1) is mathematically equivalent to the following:

(2) $$\frac{dC\left(x_{i}\right)}{dt}\propto \frac{d(ln\;x_{i})}{dt}.$$

Upon integration of Equation (2), *C*(*x_i_*) becomes proportional to *ln x_i_*. This is similar to the relationship in information theory [45], where the “information” for a two-state bit, *i*, with probability *p*(*i*), is given by log_2_[*p*(*i*)]. While Shannon’s information is not considered equivalent to complexity, both have similar scaling equations. This type of scaling is also present in the determination of the dimension in fractals as a method to yield fractal complexity (see: https://en.wikipedia.org/wiki/Fractal_dimension (accessed on 1 December 2025)).

The integration yields constants that can be represented in the usual form of a linear equation:

(3) $$C(x_{i})=aln(x_{i})+b.$$

This yields a linear relationship between *ln*(*x*) and complexity with unknowns that may be solved for. If the derivative with respect to time is retaken, then the slope, *a*, represents the rate of change in complexity as *x_i_* changes.

Complexity defined in this manner creates a unitless quantity because the logarithm of a number has no units. This allows summation over features of complexity obtained from measurements with different units. This results in an overall complexity representing the order of magnitude changes with time.

For this work, eight organism sub-systems have been identified with supporting data. These will be treated as complexity components along with a major feature for each component (see Table 2). Features have been examined using *log x* versus time plots to capture the trends in complexity (parameter *a*) since life appeared on Earth. Using the slopes obtained from regression fits to Equation (3), the trends are extrapolated to earlier times toward the era when one of the simpler forms of the complexity feature might have begun its evolution. These projections are shown in the Results section. The selected endpoints are given in Table 2.

An easy-to-understand example is first presented using the complexity growth of computer components over several decades. It shows the linear relationship between log(*x*) and time for function, structure, and memory components. It also demonstrates the utility of extrapolating back in time to the earliest forms of complexities. This example also shows that plotting complexity growth vs. complexity growth yields the equivalent of an allometry-style plot. With the addition of a suitable time “ruler”, such log–log plots become useful for extrapolating back to earlier times.

## 3. Materials and Methods

### 3.1. Data

**Figure 1:**
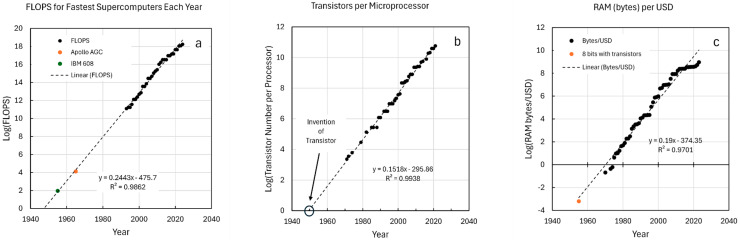
Trends in computer FLOPS, transistor counts, and RAM per USD are rendered as log-linear plots versus time. Data for all three plots were obtained from Our World in Data website [64,65,66]. (**a**) FLOPS were obtained in the form of GFLOPS for the fastest computers by year. This was converted to log (FLOPS) and plotted versus time. The FLOPS for the AGC [61] and IBM 608 (see: https://en.wikipedia.org/wiki/IBM_608 (accessed on 1 December 2025)) were added as individual points. (**b**) Data for transistors per microprocessors were plotted as obtained from the source. The intersection of the regression line at log = 0 was 1949.3 yr. This is approximately the time of the Bell Labs demonstration and patent of the first transistor [67]. (**c**) RAM (bytes) per USD was derived from cost per terabyte of RAM in 2020 US dollars [66] and the logs of these values were plotted versus time. The commercial price of one transistor in 1955 and conversions from 1955 dollars to 2020 dollars were obtained as approximates provided by the Google AI search engine.

For the log-linear plots of feature values versus time before the present (in Gyr), the times of various species appearances on Earth are estimated using values reported from various sources. The appearance of bacteria, archaea, eukarya, and all subsequent taxa on Earth has not been firmly established but many estimates have been proposed. In Table 3, crude estimates for the appearance of several major organism classifications are obtained from various publications and internet searches. These values were used in some of the subsequent plots of complexity features versus time of organism origination. The minimal organism, JCVI-syn3A [46,47,48], was considered a possible surrogate for LUCA and thus was positioned at approximately 3.5 to 4 Gyr before the present on the evolutionary time scale. While JCVI-syn3A may be incapable of surviving in the wild, it provides an example of a living organism with most of the components of Figure 1a. Choosing a very small natural occurring prokaryote is an alternative option. Even the stromatolite cyanobacteria could be used. The advantage of JCVI-syn3A is that it has been studied in various ways and offers data used for Section 4.5 and Section 4.6. For the other sections, alternate surrogates for LUCA do not change the analyses significantly.

The measurements on species currently alive are assumed to be tightly linked to the feature values these organisms exhibited when they evolved into a new species long ago. While epigenetic variations would have occurred over time to adapt to niches, the basic structure (e.g., DNA length, protein complement, internal network) would remain constant or, by definition, the organism would be a different species. Therefore, measurements on currently alive organisms can be placed on a time scale associated with species formation as inferred from log-linear plots or from log–log plots with an overlaid time “ruler”.

Many data sets were obtained from supplementary data provided by referenced research papers. These are denoted in the sections below and the figure captions.

**Table 3:** Origin times for various organisms.

Taxon	Gyr Before Present	Est. Mean	Sources
Archaea	3.3–3.5	3.4	Javaux [49] Noffke [50]
Bacteria	3.5; 3.5; 3.4; 3.3	3.42	Awramik [51] Javaux [49] Westall [52]
Cyanobacteria	3.6; 2.6; 2.7; 3.4 3.1; 2.5; 2.7; 2.8 3.2	3.2	Sanchez [53] Buick [54]
Red Algae	1.7; 1.5	1.6	Westall [52]
Green Algae	1.1; 1.1	1.1	Westall [52]
Fungi	2.06; 1.57; 1.1; 1.1	1.46	Bengtson [55] Loron [56] Westall [52]
Arthropods	0.48 0.53	0.5	Misof [57] Wolfe [58]
Land Plants	0.45–0.5	0.47	Cambrian Explosion Summary in Wikipedia
Invertebrates	0.65–0.55	0.6	
Vertebrates	0.45–0.55	0.5	
Mammals	0.3	0.3	

### 3.2. Analyses

For two features (Section 4.3 and Section 4.4), log–log plots are presented. These are considered complexity vs. complexity, similar to allometry or power law plots. As shown in Section 4.1, allometry-style plots can be considered complexity growth plotted versus complexity growth with time removed. The slope is the ratio of the slopes of the original complexity growth plots before suppression of the time scale. Therefore, these log–log plots can be used to extract an estimate of time progression with the addition of a suitable time “ruler” to replace the suppressed time vector. For evolution, that time ruler is scaled so that the present time is approximated with human appearance, the time point ~1.9–2.5 Gyr before the present is associated with the appearance of eukaryotic cells, and the time point 3.5–4 Gyr before the present is associated with the minimal organism JCVI-syn3A or the simplest bacteria in the particular complexity feature. Such a ruler can be extended further back in time to simpler instances of the two complexities plotted.

Log to the base 10 was chosen for all plots and was the base for all logarithms unless otherwise noted. While this does not give a value for complexity itself, it gives a relative value that is equally valid in linear extrapolations. As with Shannon’s information theory and the concept of “nats” to provide the base for the logarithm [2], there are optional and perhaps more relevant base values for each of the component features that were examined. Different bases simply scale each point on a log-linear plot, resulting in a slope change but not an intercept change along the time axis. For example, the plots of transistor count in Figure 1b can be redrawn with log base 2, to be more consistent with Moore’s doubling time, and the intercept for one transistor is still ~1949.

All analyses and figures were created in Microsoft Excel (Microsoft Corporation, Redmond, WA, USA.)

## 4. Results

### 4.1. Examples

#### 4.1.1. Computer Electronics

The development of the modern electronic computer has shown a consistent increase in complexity. Three features of this development have been chosen to demonstrate functional, structural, and memory progression over time. The Floating Point Operations Per Second (FLOPS), number of transistors in microprocessors, and the memory abundance per US dollar are shown in Figure 1a–c. Each of these are renditions of Moore’s Law [59] and remain linear in the log_10_ space over several orders of magnitude. Computations per Kwh over time for various computers also has a log-linear relationship over twelve orders of magnitude [60], but is not included in this example.

Each of these features represent components of computer complexity and show the expected linear increase in log(x). The regression lines have been extrapolated to time points before the beginning of the primary data. In the case of FLOPS (Figure 1a), the speed of the Apollo Guidance Computer falls directly on the line. This was the first use of integrated circuits in a stand-alone computer [61]. The IBM 608 was the first commercial calculator made with transistors (see: https://en.wikipedia.org/wiki/IBM_608 (accessed on 1 December 2025)). Its FLOPS rate also falls on the line. In the case of transistor number in microprocessors (Figure 1b), the extrapolation to Log = 0 (i.e., one transistor) intersects the date when the transistor was invented and patented [59]. This was noted by Moore [62], and it demonstrates the utility of this relationship as a complexity plot. In the case of the cost of RAM (Figure 1c), the number of bytes that can be purchased for a dollar (adjusted to 2020 dollars) was extrapolated back to the time when transistors were first available commercially (1954). Eight bits (one byte) would require 16 transistors at $10 apiece in 1954 money, which would be $100 apiece in 2020. So, the number of bytes that could be bought for 1$ would be 1/1600. The log of this number is −3.2 and is shown by the orange dot in the figure.

In all three figures, the plots extrapolate backward in time linearly to the earliest prototypes of the respective features that provide the bases for the modern, ever evolving computer. It is also worth noting that computers based on microprocessors located anywhere along the regression line could still be operating today. They may have been adapted to provide more capabilities with increased memory, expansion boards, and overclocking, but their fundamental place in history is their location on the line. When placing modern organisms in history, it is assumed that their origin belongs where their complexity aligns with the trend.

**Figure 2:**
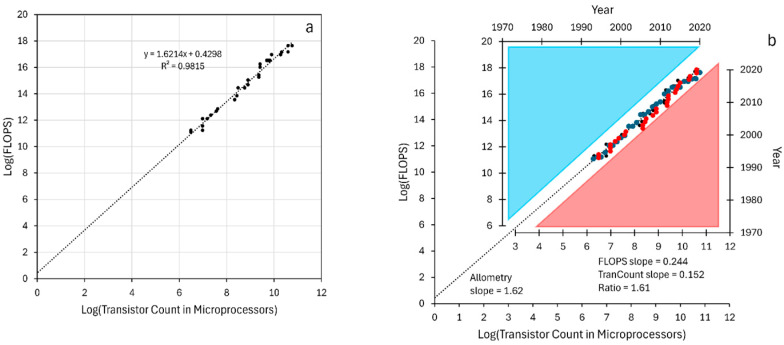
FLOPS and transistor counts rendered as a log–log (allometry-style) plot. (**a**) Log of FLOPS were plotted versus log transistor count per microprocessor for the years common to both sets of data. The linear regression information is included in the figure. (**b**) The axes from Figure 1a,b are inserted into the log–log plot. The axis containing the blue triangle and blue data points is log [FLOPS] vs. time. The axis containing the red triangle and red data points is log [transistor count] vs. time. Each axis has been reoriented to allow the data points to best align with the log–log plot of panel A. The slope of the log–log plot can be derived from the ratio of the log vs. time plots.

The allometry-style plot (function vs. “mass”) of Figure 2a contains the information of Figure 1a,b with time removed. In Figure 2b, the plots of Figure 1a,b are shown with axes reoriented to allow the data points to lie along the log–log plot. The slope in the log–log plot is simply the ratio of the slopes for each complexity growth plot, as shown in the figure. One can extract the log–log plot slope and consider it a power exponent [25,63] that relates the two variables under the assumption they are interdependent. That approach hides the fact that the slope is fundamentally related to two complexity growth rates. It also hides the possibility that they might not be related to one another. Plotting the RAM per dollar (Figure 1c) versus transistor number would yield a linear log–log plot, but their interrelationship is convoluted, at best.

More importantly for this work, the dimension reduction can be partially reversed to yield a time surrogate vector when times are not known well. For biological evolutionary data, each pair of data points in an allometry-style plot has a common time point that is missing and likely unknown. Nevertheless, the monotonic progression of data pairs in the log–log plot predicts a monotonic progression of time associated with the points. Therefore, a time “ruler” can be applied to the log–log plots using known alignment anchor points, as mentioned in Methods. The ruler can give approximate times for the complexity values.

#### 4.1.2. Additional Examples—See Appendix A, Appendix C and Appendix D

Appendix A

The growth of global energy usage over time follows a log-linear relationship, indicating that it is growing exponentially. Viewed in the context of complexity, the logarithm is growing linearly with a sudden transition between 1950 and 1970. After the transition, it continues growing at the same rate as before the transition. This would appear to be an example of the complexity jump predicted by Nehaniv & Rhodes [40].

Appendix C

Similarly to computer electronics, the growth of the US Library of Congress holdings and the growth of US population are shown to exhibit log-linear complexity characteristics. The log–log plot, with time removed, exhibits the same slope relationship to the log-linear plots, as demonstrated for computer features.

Appendix D

Two hierarchical levels of complexity are shown for the Rubik’s cube puzzles for the transitions across 2 × 2 × 2, 3 × 3 × 3, and 4 × 4 × 4 sizes. Extrapolating each hierarchical level to a 1 × 1 × 1 cube yields the expected trajectory length for moving along its faces. This example demonstrates both the extrapolation to simpler complexity and the behavior of features when complexity takes a hierarchical jump. Many biological features, like DNA, RNA, and protein structures have extremely high levels of combinatoric complexity, similar to the possible configurations of a Rubik’s cube. However, working at an intermediate level of complexity for biological systems provides a strategy to simplify comparisons across components of complexity.

### 4.2. DNA Nucleotide (nt) Length

A strand of cellular DNA has a massive capacity to store information. Therefore, it has a huge complexity potential. A DNA string in a living organism, however, has collapsed that capacity to an instance of complexity. When more information needs to be stored, it is not erased and reprogrammed so as to use all of its complexity potential; rather, it is simply elongated. The evolution of species has used this mechanism to create more complicated organisms over the course of the 4 billion years life has existed on Earth. Therefore, the increase in capacity is proportional to the increased DNA length and hence Equation (2) applies, with x representing the number of nucleotide (nt) base pairs in an organism’s DNA.

**Figure 3:**
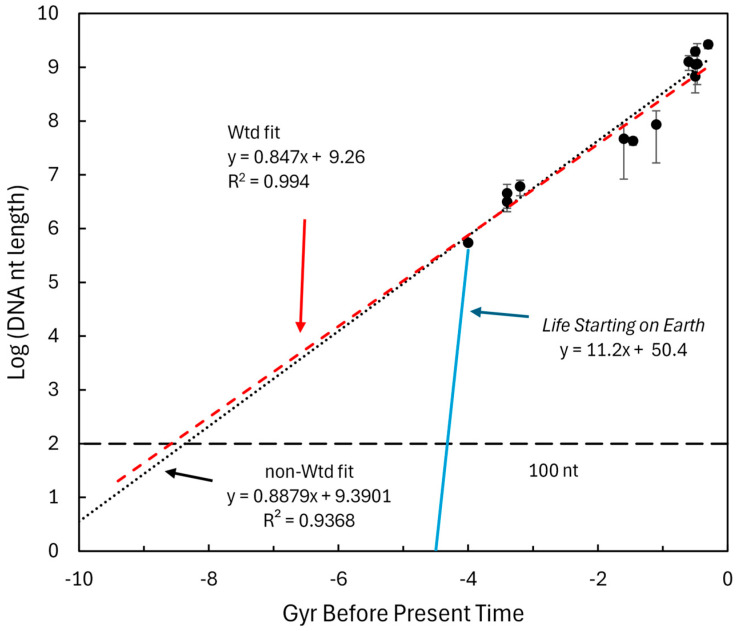
Log of genome length in nucleotide pairs (nt) versus approximate time for the origin of various taxa. A total of 25,987 samples were extracted from NCBI where both genome length and gene number were reported for the taxa of archaea, bacteria, cyanobacteria, green algae, red algae, fungi, arthropods, land plants, fish, birds, reptiles, and mammals. The genome length for the artificial minimal organism JCVI-syn3A was included as a surrogate for LUCA and assigned a time of origin of 4 Gyr before the present time. Vertical error bars represent the ±one standard deviation of the genome lengths in each taxa and were then converted to logarithms. The non-weighted and weighted regressions are shown with regression information. Since the bacteria collection constituted 21,318 entries, it was excluded from the weighted regression. The blue line represents the growth of complexity necessary if pre-biotic life and early life occurred only on Earth.

Genome nt length was extracted from NCBI. The plot of log(nt) versus organism appearance before present time is shown in Figure 3. The minimal feature chosen (Table 2) was the DNA length needed to code a single peptide of 33 aa (i.e., 100 nt). While this cannot be considered representative of the earliest chemistry of life, it would be a hypothetical early signpost on the path to FUCA, representing the potential amount of complexity in molecules at that time. The intersection of the regression line with the 100 nt level of complexity is approximately 8.4 Gyr before present time.

A blue line is added to Figure 3 to represent the dominant conjecture that all pre-biotic chemistry occurred on Earth. Implicit in this conjecture is that complexity grew very rapidly until life formed and then shifted to a slower growth during evolution.

The data extracted from NCBI was sufficient to generate fairly robust variation in DNA content for each taxon at its estimated time point. This allowed a Monte Carlo analysis to determine the overall variance of the slope and intercept. The analysis constituted 1000 random instances where the standard deviation for each taxon was used to define a normal variate for DNA nt length at each point. The variation in the time dimensions was defined as ±20% at each time value and these values were also drawn from a normal distribution. The resulting coefficients of variation for the slope and intercepts were 8.5% and 8.0%, respectively.

### 4.3. Number of Genes

A total of 2165 eukaryotic entries for genome length and gene total were extracted from the NCBI database spanning mammals, reptiles, fish, land plants, birds, arthropods, fungi, and red and green algae. A total of 22,323 prokaryotic entries were also obtained spanning cyanobacteria, archaea, and other bacteria.

**Figure 4:**
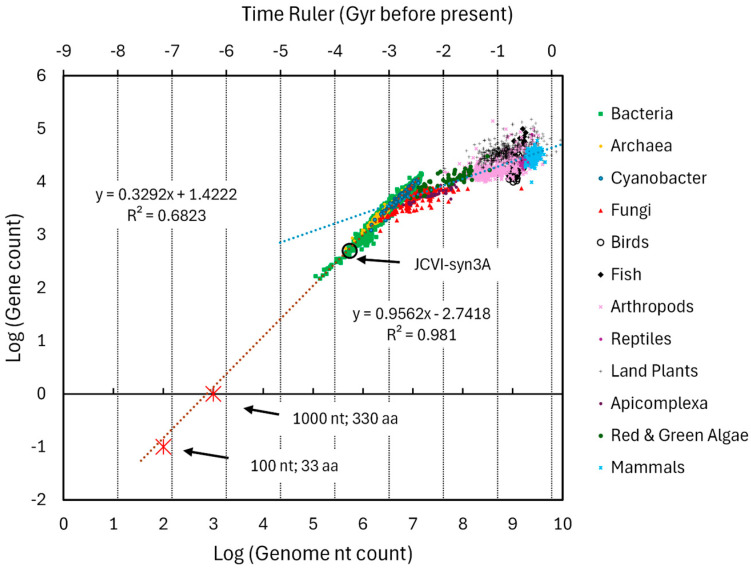
Log of total gene count versus log of genome length (in nt) for each entry spanning archaea to mammals. Taxa colors are shown in the legend but, considering the overlap, it is difficult to distinguish many of the taxa. Vertical gridlines are associated with the time ruler.

In Figure 4, the log of total genes was plotted versus log of genome length (in nt) for each entry. Two slopes are readily evident. The prokaryotes (bacteria, archaea, and cyanobacteria) defined a tight line with a high regression coefficient and a slope of 0.96. The collection of eukaryotic data points defined a much shallower slope of 0.32 and a regression coefficient indicating significant scatter. A time ruler was aligned to the data placing current time just after the last cluster of points, the region around −2.5 Gyr near the transition between prokaryotes and eukaryotes, and the region between −3.5 to −4 Gyr near the single entry for the minimal cell JCVI-syn3A. The lowest values on the bacteria locus of points tend to be endosymbionts and are not representative of organisms that can survive outside of another organism. Thus, a better position for the alignment of the −3.5 to −4 Gyr point on the time ruler was JCVI-syn3A.

Assuming a single prokaryotic protein would have a length of ~330 aa generated by a string of ~1000 nucleotides, this can be plotted at the intersection of log(gene) = 0 and log(DNA nt count) = 3. This is shown in the figure as the upper of two red stars. The lower star represents a ~33 aa peptide generated by ~100 nt. Both of these are close to the regression line, as would be expected for organisms that have no introns or few non-coding regions.

At the same point chosen for Section 4.2 [100 nt, 33 aa], the time ruler indicates approximately −7.2 Gyr before the present time. Given the uncertainties of the time ruler and the fixed time values in Figure 3, this is generally consistent with the extrapolation of nt length plotted directly against evolutionary time estimates in Figure 3. This provides simultaneous gene and genome evidence for very early precursors of low complexity being located on the complexity growth curve well before the Earth existed.

The ratio of slopes method, demonstrated in Section 4.1.1, can generate the rate of complexity change with time for gene number for the prokaryote locus of points. Therefore, the product of the slope in Figure 4 with the slope of Figure 3 yields a complexity growth rate for gene number equal to 0.96 × 0.81 = 0.78 Gyr^−1^.

**Figure 5:**
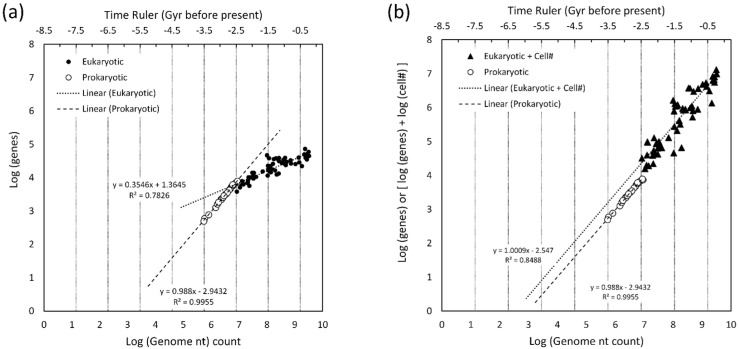
(**a**) Log of total genes versus log of genome length (in nt) for each entry spanning archaea to mammals. (**b**) Log(genes) plus log(# cell types within an organism) versus log of genome length (in nt) for each entry spanning archaea to mammals.

For eukaryotes, the locus of points has a much lower slope, indicative of slower growth in gene number with increased genome length. The facile conclusion for this would be that growth of complexity shifts from gene creation to the complexity associated with multicellular organisms. This conclusion is examined more closely using data collected by Alvarez-Ponce & Krishnamurthy [22], primarily consisting of data published by Schad et al. [68] and Vogel & Chothia [69]. Alvarez-Ponce & Krishnamurthy examined complexity, as defined by the number of unique cell types in an organism, versus various aspects of the proteome (genes, families, clans, domains, and motifs). Their data spanned prokaryotes to eukaryotes for 79 cases where all the information is known. Using their data and reproducing the format of Figure 4, the log–log plot for genes versus genome length is shown in Figure 5a. This closely reproduces the distribution of data points in Figure 4 derived from a larger NCBI retrieval and generates very similar slopes.

Under the first hierarchical complexity axiom of Nehaniv & Rhodes [40], which states that complexity is additive, the sum of log(genes) and log(# cell types within an organism) was created as a combined feature of complexity for eukaryotic cells. This is shown by the triangles in Figure 5b. The regression of this set of points closely matches the regression line of prokaryotes. Upon re-introducing time, the interpretation is that the complexity growth rate for prokaryotic genes and eukaryotic genes + differentiated cell number are the same. So, the relentless growth in complexity can shift from one type of structure to another as the need for lower level features (genes) is diminished.

Once complexity growth is linearized and harmonized across prokaryotes and eukaryotes, a second time ruler could be introduced along the vertical axis, similar to Figure 2b. This would provide two estimates of time for the origin of each species in the data set, but that is outside the scope of this paper.

### 4.4. Protein–Protein Binding

**Figure 6:**
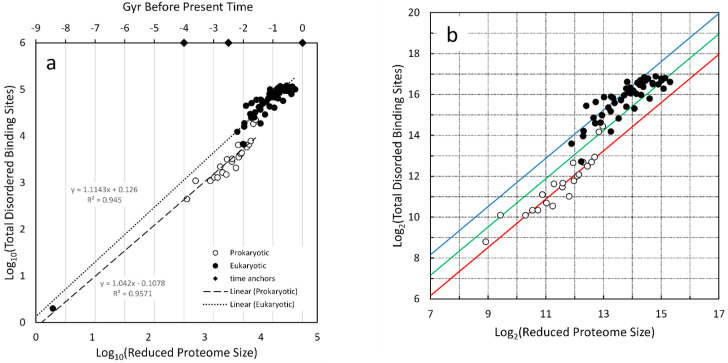
Log of total disordered binding sites in proteins versus log of the reduced proteome size. These values were collected by Shad et al. [68]. The reduced proteome size was determined by eliminated gene sequences that produced the same protein. (**a**) Regression lines were calculated separately for prokaryotes and eukaryotes on the log10 vs. log10 plot. These were extended to the data point for two proteins and two binding sites. A time ruler was applied with the times for JCVI-syn3A, the transition to eukaryotes, and current time represented by diamonds. The approximate time for [2 proteins, 2 binding sites] is 8.5 Gyr before the present. (**b**) The same data as panel *a* was plotted on a log2 axes. The red, green, and blue lines are separated by unit log values, i.e., by factors of 2. These were chosen arbitrarily to visually match the locus of points.

Schad et al. [68] examined proteome size, protein structural disorder, and organism complexity as measured by the number of unique cell types within an organism. The cell number collection for organism complexity was used previously in Section 4.3. Here, the data for disordered binding sites and proteome size, derived from their supplementary Table S1, is used in the log–log plot of Figure 6a. Two regressions are noted, one for prokaryotes and one for eukaryotes.

Both slopes extrapolate to the simplest form of protein–protein interaction, which is a point at the lower left indicating two proteins and two disordered binding sites. According to the time ruler, that is approximately 8–9 Gyr ago. Here, again, the idea that full proteins exist at this early time seems unrealistic. Rather, this is another signpost that signifies the complexity associated with two amino acid sequences, probably derived from inorganic synthesis, having structures that can allow non-chemical binding to each other. This canonical form of binding provides the underpinning for the evolution of protein–protein networks.

There is a clear transition between prokaryotes and eukaryotes (Figure 6a). To visualize this more easily, the logarithms in both dimensions were converted to log_2_, thus highlighting factor-of-two jumps (Figure 6b). The character of the transition is similar to the jump seen in the progression of global energy use (see Appendix A). The theoretical possibility of such a jump was predicted by Nehaniv & Rhodes [40]. The red, green, and blue lines are not regressions but were added to help visualize factor-of-two jumps. There is a factor of four in the increase from prokaryotes to eukaryotes indicative of two 2-fold jumps. The slopes before and after the transition are very similar, indicating complexity continues to grow at approximately the same rate (Figure 6a) even after a 4-fold increase at the transition. Niklas et al. [70] examined the role of intrinsically disordered proteins in the evolution of cells and also noted the jump in such disordered domains in eukaryotic cells.

The ratio of slopes method, demonstrated in Section 4.1.1, can generate the rate of complexity change with time for disordered binding sites. Therefore, the product of the prokaryote slope in Figure 6a with the value calculated for gene number in Section 4.3 yields 1.04 × 0.78 = 0.81 for the rate of complexity increase. Using the slope for eukaryotes in Figure 6a, the result is closer to 0.87.

### 4.5. Energy to Build a Cell

**Figure 7:**
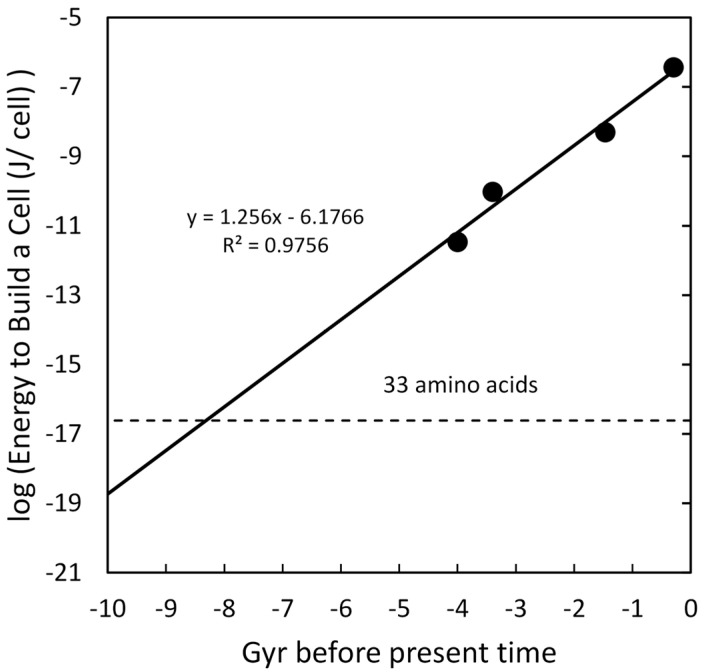
Log of energy to build a cell versus time of organism origination. Variability reported by the authors is 9.37%, 15.92%, 16.13%, NS 12.68% for mammals, yeast, bacteria, and JCVI-syn3A, respectively. After taking logs, the range of these error bars are smaller than the data points in the figure. The horizontal dashed line is the log of the energy to build an average amino acid in J/molecule.

Ortega-Arzola et al. [71] undertook the challenge of calculating the energy required to assemble a cell from known components. They gave values for mammal, yeast, bacteria, and JCVI-syn3A cells. The logs of these values are plotted in Figure 7 versus approximate time for the origin of these lifeforms. With only four points, it is difficult to be confident of the slope but the regression coefficient is reasonable enough to suggest the simplest interpretation of a linear relationship. The targeted early feature was the average energy for amino acid synthesis [72] in ATPs for an H2-dependent methanogen obtaining nitrogen from NH4+ or N2. The average of ATPs required across both nitrogen sources was 11.65. This was converted to Joules for the plot. The resulting intersection of the regression line with the energy to build 33 amino acids was at ~8.3 Gyr before the present time.

### 4.6. Volume (Mass) of a Cell

**Figure 8:**
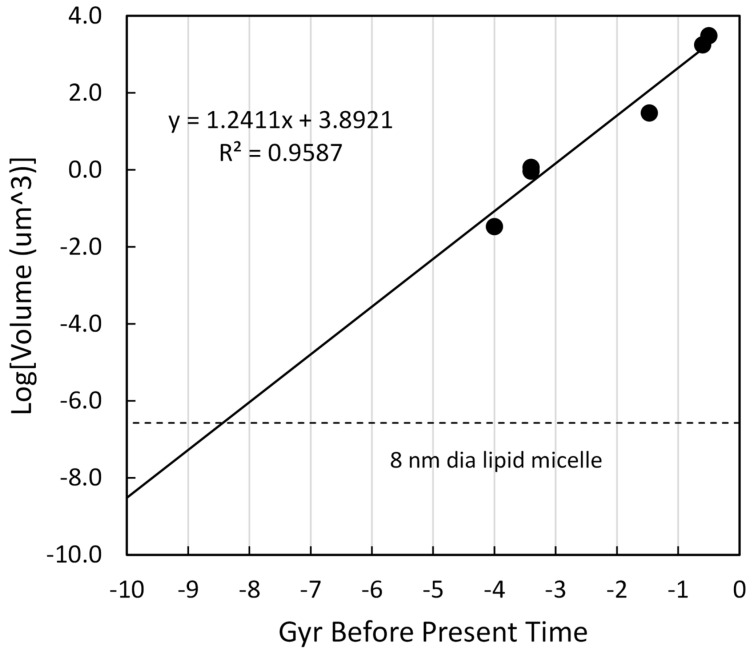
Log of organism cell volume versus time of organism origination. The diameter of a 33 amino acid peptide was provided by the online calculator at https://nanocomposix.com/pages/molecular-weight-to-size-calculator (accessed on 3 January 2026). This was given as 2.0 nm. It was assumed this was encased in a micelle with wall thickness of 3 nm, generating a micelle diameter of 8 nm and a volume of 2.68 × 10^−7^ µm^3^. The log of this value is shown in by the horizontal dotted line.

Cell size is fluid and under the control of many factors during growth, DNA replication, division, and stasis (see: [Amodeo & Skotheim, [73]) but the vast differences between bacteria and vertebrates still allow the use of estimates. Milo [74] defined the canonical values for cell volumes, in µm^3^, as 1.0 for *E. coli*, 30 for budding yeast, and 3000 for HeLa cells. The *E. coli* estimate is used for bacteria and archaea, the yeast estimate for fungi, and the HeLa estimate for vertebrates. The value of 1767 µm^3^ for invertebrates was derived from an estimated diameter of 15 µm, which lies in the middle of the range often given in public domain searches. The volume of JCVI-syn3A was obtained from the reported diameter of 0.4 µm [47]. These values are plotted versus time before the present in Figure 8. The regression slope is 1.27 with a coefficient of regression of 0.97.

The complexity regression line intersects the volume of a 33 amino acid peptide, encased in a lipid micelle, at approximately 8.5 Gyr before the present time. In a manner similar to other arguments, this is not assumed to be an actual biological construct. Rather, this is following the theme of selecting intercept points targeting the complexity of 100 nt or 33 aa constructs. In this case, the smallest lipid enclosure to hold such a construct would be approximately 8 nm in diameter.

### 4.7. Metabolic Rate of a Cell

The metabolic rate of cells is a function of several factors, including temperature, fuel abundance, activation enthalpies of various reactions, and the nature of oxidant molecules. This makes it difficult to treat this topic in the same manner as the other features examined in this paper. Nevertheless, it seems important to include this among biological complexity components. West et al. [75] has shown that metabolic rates for mammalian cells, mitochondria, and respiratory complexes have an allometric scaling relationship with the mass of components, cells, and organisms. The slope of that relationship is approximately 0.75. Knowing the slope for the growth of mass complexity (Section 4.6), the growth of complexity for the metabolic rate can be obtained from the ratio. That gives a value of 0.9 per Gyr. Without having suitable anchor points for the time axis, nor knowledge of chemical and environmental conditions, extrapolation to early times is impractical. However, comparing the complexity growth slopes for the energy to build a cell (slope = 1.36 per Gyr) versus the energy to run a cell (slope = 0.9 per Gyr), building a cell becomes more energy-intensive with time but running a cell becomes more energy-efficient over time in comparison. One might interpret this as a trophic force leading to multicellular organisms, since individual cells of increasing complexity become harder and harder to build. Once the jump is made to multicellular structures, increases in metabolism complexity can occur through intricate intercellular functioning rather than just intracellular metabolism.

### 4.8. Growth Rate of Mass

**Figure 9:**
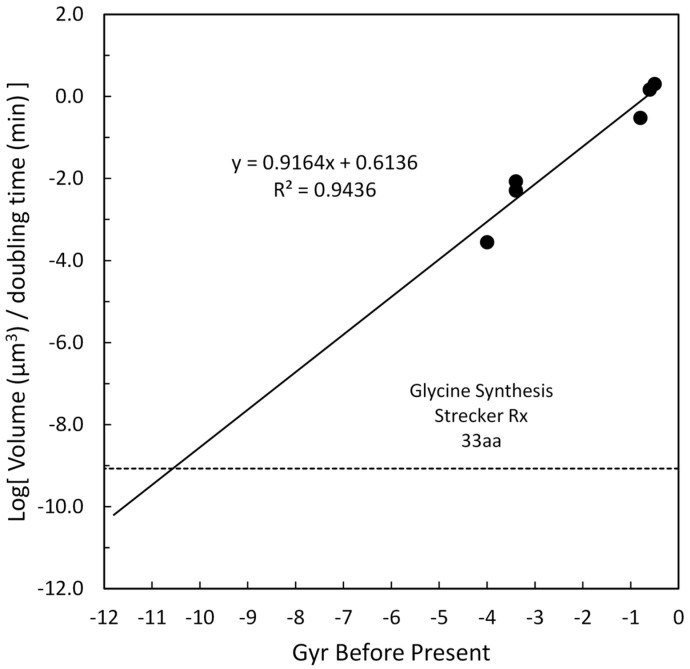
Log of volume per doubling time versus Gyr before present time for cells from various taxa. The volume per doubling time for glycine has a value of 2.56 × 10^−11^ µm^3^/min. The log of this value is shown by the horizontal dashed line.

Similarly to the motivation for including metabolic rate in Section 4.7, it was considered important to include a kinetic feature which would address the dynamics of organism growth. Cell doubling times for various taxa were approximated and, using the volume of cells from Section 4.6, the ratio of cell volume to doubling time was calculated as a surrogate for the rate of cell growth through replication. These values are plotted in Figure 9. For vertebrates and invertebrates, estimates of doubling times were 30 h and 20 h, respectively. For bacteria, archaea, and JCVI-syn3A, the published values of 3 h [76], 2.2 h [77], and 2 h [46] were used, respectively.

The regression slope was 0.93 with a regression coefficient of 0.95. Extrapolation of this regression was to the Strecker Reaction [78] for inorganic synthesis of the simple amino acid, alanine, which would be an example of a very early building block for life. Cowan [78] argued that a possible half-time for this reaction on early Earth would be about 0.3^−1^ min provided there was formaldehyde present to facilitate the final hydrolysis of the nitrile intermediate to carboxylic acid. The volume of alanine was calculated to be ~4.6 × 10^−11^ µm^3^. The ratio of volume to doubling is shown as the horizontal line in Figure 9 for synthesizing 33 aa in sequence. The intersection of this line with the complexity slope occurs at ~10.5 Gyr before the present time, which makes it consistent with the idea that simplicity precedes complexity, since this is the smallest (simplest) of all the chosen early features.

### 4.9. Molecular Complexity Index (mcbit)

**Figure 10:**
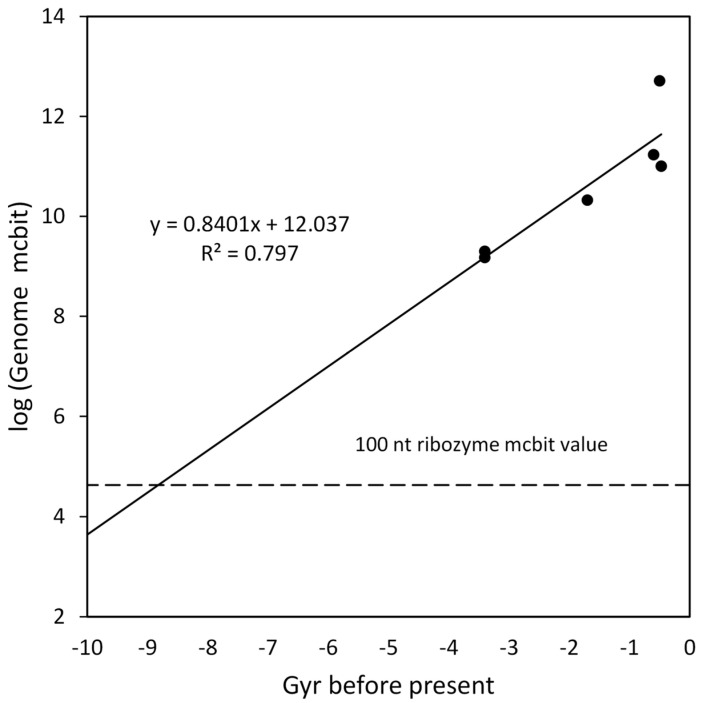
Log of genome mcbit versus Gyr before present time for DNA from various taxa. The average mcbit for a 100 nucleotide RNA molecule is 42,500. The log of that value is shown as the horizontal dashed line.

Bottcher [18,19,49] defined a molecular complexity framework and an index that could be calculated for molecules ranging from simple to complicated. He provided mcbit values for a small set of organisms and collections of molecules in his supplementary tables. A selection of these were examined in a plot over the time of organism origination. The log of genome mcbit versus time for examples of vertebrates (*H. sapiens*), invertebrates (*C. elegans*), land plants (*A. thaliana*), fungi (*S. cerevisiae*), bacteria (average of 11), and archaea (average of 5) are shown in Figure 10. The extrapolation intercept was the average mcbit value for a 100 nt RNA molecule. The intersection at ~8.8 Gyr before the present time is similar to other intersections for similar small molecules, as shown collected in Table 4, along with the complexity growth slopes.

### 4.10. Complexity Intercepts with Respect to Star Formation

The compilation of results is shown in Table 4. The complexity slopes are similar to each other with a mean (sd) for the eight features of 0.96 Gyr^−1^ (0.20). The intercepts with low-complexity features are all before the solar system formed, with the oldest being the inorganic catalysis of 33 alanines and the youngest being the formation of a memory chunk that could code a small peptide considered a gene product. The mean (sd) of the intersections is 8.6 Gyr (1.0). Five of the intercepts cluster closely together with a mean (sd) of 8.6 (0.23), representing a coefficient of variation of only 2.9%. The other two intercepts are not comparable in kind. The oldest represents the reaction rate for the formation of 33 alanines sequentially but does not take into consideration the rate of peptide bond formation. The youngest represents the formation of a 33 aa peptide as derived from a plot of genes versus genome. This intrinsically assumes a mechanism already exists to translate one from the other. That is an assumption that is not shared by the other complexity trend lines.

In Section 4.2, a Monte Carlo simulation was described that calculated the variation around the slope and intercept for the plot of DNA length versus time. These variations are less than ten percent, which supports an interpretation that linear complexity growth could have an origin around 8–9 Gyr before the current time. The other features examined had limited cases and a similar Monte Carlo simulation was not practical. The values reported for the energy to build a cell, Section 4.5, did include estimates of variance (see Figure 7 caption), but these were significantly smaller than the variation in DNA length of Section 4.2, so the anticipated slope and intercept coefficients of variation would be less than ten percent. As more data become available, the statistical significance of many of these projections back in time can be evaluated. For now, the aggregate consistency is a phenomenon worth some attention.

**Figure 11:**
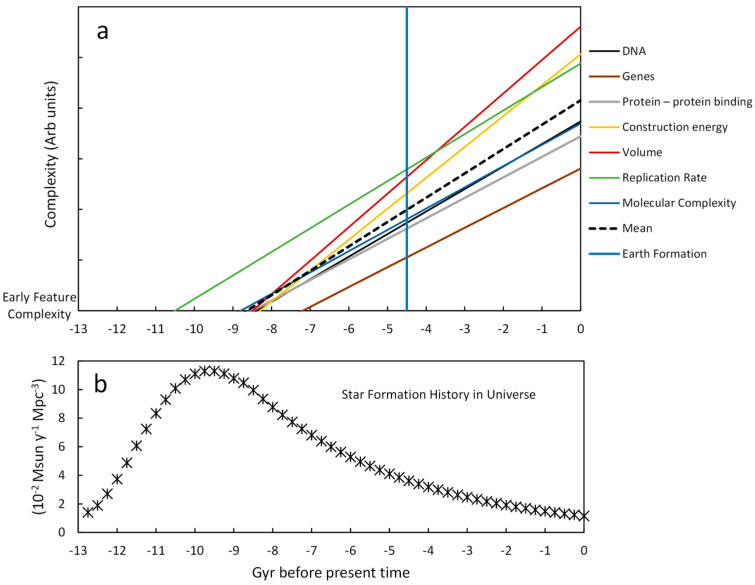
Alignment of complexity slope intercepts with star formation in the universe. (**a**) Lines were generated with the slopes and intercepts at early features from Table 4. All intercepts are placed at the “Early Feature Complexity” level of the figure. The mean line was generated from the mean slopes and mean intercepts of Table 4. (**b**) Star formation data points were generated as described in the text and in the Supplementary Information. The vertical blue line represents the approximate time of Earth formation.

A compilation of the slopes and the intercepts at early feature levels is shown in Figure 11. It reveals that the amount of complexity growth during life on Earth is preceded by an almost equivalent amount of complexity growth prior to Earth formation under the assumption of linear complexity growth. The vertical line at −4.5 Gyr denotes the earliest time when water likely appeared on Earth and it lies approximately halfway to the average intercept of 8.6 Gyr before the present.

The bottom panel of Figure 11 denotes the rate of star appearance in the universe as determined by Ajello et al. [79] (their supplementary Table S5), measuring the attenuation of gamma rays by extra galactic background (EBH) light. The original data (Extragalactic Background Light (EBL) vs. red shift, z) was fitted with a 4th-order polynomial function from the big bang to the peak and an exponential decay function after the peak. These were combined and sampled every 0.2 Gyr for plotting. The red shift, z, was converted to Gyr after the big bang using the online calculator by Edward Wright (see: https://www.astro.ucla.edu/~wright/CosmoCalc.html (accessed on 11 May 2025)). This was converted to time before the present by the subtraction of 13.721 Gyr, which is considered the age of the universe. The authors’ model provided EBL conversion to star appearance, which is shown as 0.02 times the formation of stars with the mass of the Sun per year per megaparsec. More details are given in the Supplementary File for the construction of Figure 11.

The two panels of Figure 11 are aligned to demonstrate that the number of stars, and therefore planets, was highest when biological complexity might have begun its growth. This cannot be considered cause and effect but simply represents a phenomenological concordance worth noting.

## 5. Discussion

### 5.1. Recap and Comments

A take-away from this analysis is that complexity growth over the last 4 Gyr has been approximately linear for many features of Earth’s organisms. If one assumes that the observed linear growth is the continuation of an unseen and unmeasurable linear growth preceding it, then that leads to the conclusion that life started developing somewhere else several billion years before Earth was formed.

The two conjectures considered here for the timing of the origin of life state that either complexity grew linearly from pre-biotic chemistry to life today or that complexity grew at different rates for pre-life and life itself. In the spirit of Occam’s Razor, the simplest solution would be a complexity growth rate that remained constant from the start of pre-biology chemistry and throughout life today. This paper is embracing that simplest solution as the first best choice. It neither attempts to prove anything or even reach a point of providing a testable hypothesis. It is a phenomenological approach. Such approaches usually precede hypothesis creation by providing interesting relationships among observations that may need further study.

One concern about declaring that the growth of complexity is linear derives from the controversial reports of evolutionary jumps, commonly referred to as punctuated equilibrium (see Pennel et al. [80] for a discussion). One can compare such jumps in evolution to saltatory growth which looks like stepwise changes up close, but looks smooth when viewed over long periods of time. Using physics terminology, what is the scale length (length scale) that represents our phenomenon of interest, i.e., complexity changes? The conjecture explored in this paper envisions linear complexity growth on average, where the scale length is many millions of years. Only a large jump, like the one between prokaryotes and eukaryotes, breaks the average linear complexity growth for certain features because it is detectable at the scale length used in this analysis.

Another concern is how one justifies extrapolating so far back in time. This concern rests in the assumption that organism evolution derives from natural selection and this only works for living organisms. I think it is worth considering that there is more to evolution than natural selection. Is it possible that complexity breeds complexity? If so, the growth of complexity in pre-biotic chemistries does not have to respond to natural selection. It simply has to be self-selective. A chemical reaction can “compete” for resources simply by having a faster reaction rate for the reactants available. This process favors certain reactions, certain autocatalytic chains, the building of a limited set of polymers, the formation of certain catalytic polymers, etc. I suspect that it is not a fast process, which is why I find the many billions of years predicted by the linear extrapolations appealing.

It should be noted that the plots in this paper have not accounted for the duration needed for Sun and planet formation, which has been estimated to be between 0.1 and 0.2 Gyr [81]. During this time, life within the solar nursery nebula would not be evolving and hence there would be a discontinuity in the plots presented. Such a discontinuity would be small and well within the expected variance of all the points being plotted.

In the early phases of the Earth’s history, there were faster days and shorter months altering the “ticks” of time as measured by the day/night cycle. (Years, themselves, would have been unaffected.) Tides would also be higher and more frequent since the moon was closer to Earth. Would these faster cycles be sufficient to introduce all the complexity of life in Earth’s early period? Without knowing the actual steps for life’s origination, it is difficult to determine this. This is left as an open question. However, a few hundred million years of faster days is not enough to maintain linearity in the plots presented in this paper if one is forced to move the minimum of complexity to a starting point around 4.3 Gyr ago or when the planet cooled enough for liquid water.

An important perspective on the growth of complexity is that it seems slow in the log scale but that should not be confused with the growth of the features examined. While a complexity slope of 1 Gyr^−1^ means it takes a billion years for complexity to grow by one unit, the feature count has increased by a factor of 10 in that time. Furthermore, total complexity is the sum of component complexities. The number of components, while not known, could be substantial. Therefore, the growth of total complexity could be significant each billion years. In addition, the number of combinations among feature values is the product of the features values for each component, not the sum. This grows at a steep exponential rate. It is also worth noting that the unit of complexity has not been defined. Log10 is generally not suspected to be the optimal base for each feature, but it is used for convenience. The explorations of the “k-mers” or “nats” used in Shannon’s information theory [2] may provide insight into the appropriate bases to choose.

Deciding what is complexity and what is not seems to generate problems in biology. The results of this analysis, particularly Figure 5b, appear to address the G-value paradox [82], which is the moniker given for the lack of correlation between gene number and organism complexity, where complexity is considered the number of differentiated cell types within multicellular organisms. This paradox was preceded by the C-value paradox [83], which addressed a lack of correlation between genome size and complexity. In the context of the definition of biological complexity presented here, both of these paradoxes basically evaporate. This is because gene number and the number of differentiated cells appear to combine into a single complexity and one should not try to predict one from the other. To do so is to ask the wrong question.

### 5.2. Implications and Predictions

If pre-biotic chemistries and early life started 6–10 billion years ago and seeded the solar nebula, then two predictions are easy to draw:

1: All evidence of life in our solar system will be RNA, DNA, triplet code, protein, membrane (RDTPM)-based.

2: We should look for similar RDTPM life in stars formed within the same nebula as Sol [84].

Other implications are that, if the Sun’s condensing nebula was seeded with the same form of life, then Earth would have received multiple “doses” of life during early formation and all would be of the same RDTPM form. This mitigates the problem of depending on only one injection of an organism at one point in time to survive a harsh environment or one extremely rare series of events to start life on Earth de novo.

Mars did not lose its water until around 3B yr ago. If it was seeded at the same time as Earth, there should be plenty of evidence of early life in appropriate locations on Mars. Lower temperatures may have made it grow more slowly before the planet lost water so its growth and spread may have been slower. That life should be of the same architecture as Earth’s. The icy moons of Jupiter and Saturn may also be repositories of early forms of life with RDTPM architecture.

Bacteria and archaea architectures may have arisen from two different organisms seeding the solar system. There is no reason to assume that all life thrown into a nebula would be of exactly the same architecture, without some minor differences. What is required is that the lifeforms must be capable of surviving deep freezing for long times, thus favoring tough single-cell organisms.

Moving the origin of life to another time and to other planets (or moons) frees us from the limitations of the conditions of early Earth. This opens the possibilities of more friendly, or harsher, conditions that could allow the chemistry of life to begin more readily.

Finally, an underlying theme of the results presented here is that biological life may be the product of continuous and unrelenting complexity growth. It invites the question of what could drive such continuous complexity growth. One might argue that one complexity feature of the universe (star formation rate) is decreasing during the whole time that the complexity of life is increasing. However, the average complexity of the universe is probably increasing because the death of stars continually increases the many elements heavier than hydrogen. These elements can support a combinatoric array of chemistries, many of which could be autocatalytic. Therefore, the formation of life and growth of life could be driven by a continuous stream of autocatalysis [21]. The slow selection process of pre-biotic chemistry, necessary for FUCA, may be considered the step-wise evolution of interlinked autocatalytic processes.

## Figures and Tables

**Table 1:** (**a**) Non-exhaustive list of characteristics common among biological organisms. (**b**) Higher-level components.

**(a)**	
**Category**	**Components**
Memory	Information storage system Replicating system: duplication and separation for cell division
Memory-to-Function	Mechanism to read code Mechanism to convert code to functional components
Entropy	Entropy reduction mechanisms for structures Entropy reduction mechanisms for functions
Energy	Mechanisms for energy capture, transformation, storage, and usage Catalytic structures for control of activation enthalpies and entropies
Structural Assembly	Enzyme pathways to create aa, nt, fatty acids, sugars, … Enzyme pathways to create poly-aa, poly-nt, poly-sugars, …
Network	Mechanisms to create negative feedback for stability Allosteric enzymes to provide major components of feedback, communication, logarithmic dynamics, and rate limitations Parallel and hierarchical network structures
Encapsulation	Structures to create barriers to entropy, stored energy, “poisons”, … Mechanisms for selective movement of molecules (e.g., nutrients, signals, catabolites, ions, …)
Plasticity at System Level	Mechanisms to dynamically adapt to the environment (temperature, pressure, radiation, etc.) Mechanisms to repair damage—i.e., active recovery from perturbations in the system Mechanisms to survive random mutational changes without catastrophic failure
**(b)**	
**Category**	**Components**
Emergent Properties	Collection of macromolecules to create network with “purpose” and reproducible actions every time constructed System (network) that yields several independently stable states conducive to survival (stasis, energy seeking, reproduction, information transfer, locomotion, responses to environmental signals, … Communication between cells Collections of identical systems that can yield group properties (e.g., bacterial biofilms)—Communication among group members Collections of non-identical systems yield extensive group properties (e.g., human ability to alter the planet)—Communication between cells, organs, and organisms as a dominant trait Cognition and awareness

**Table 2:** Features of complexity for analysis.

Feature	Component	Early Feature
DNA length	Information Storage	100 nt string
Gene number—and reduced proteome size	Function from memory	One 33 aa peptide
Protein–Protein binding (Disordered binding sites)	Network complexity	One pair of bound proteins
Energy to build a cell	Structural Assembly	Energy to build 33 amino acids
Volume (mass) of a cell	Surrogate for environmental reaction space	Volume of 33 aa peptide in lipid micelle
Metabolic rate of a cell	Energy needed for life	NA ^‡^
Cell vol vs. Doubling time	Growth rate of mass	Strecker Rx rate for abiotic creation of 33 amino acids
mcbit	Direct molecular complexity	mcbit value for 100 nt RNA string

^‡^ Not applicable.

**Table 4:** Compilation of complexity growth rates and intersections to early features.

Feature	Complexity Slope (Gyr^−1^)	Early Feature	Intersection Time (Gyr Before Present)
DNA length	0.89	100 nt DNA string	8.4
Gene number	0.78	One peptide	7.2
Protein–Protein binding	0.81	One pair of bound proteins	8.5
Energy to build a cell	1.22	Energy to build 33 amino acid	8.3
Volume (mass) of a cell	1.32	Vol 8 nm diameter micelle	8.5
Metabolic rate of a cell	0.90	NA	NA
Cell vol vs. Doubling time	0.93	Strecker Rx for abiotic creation of 33 glycine	10.5
mcbit	0.84	Avg nt mcbit value	8.8

**Figure A1:**
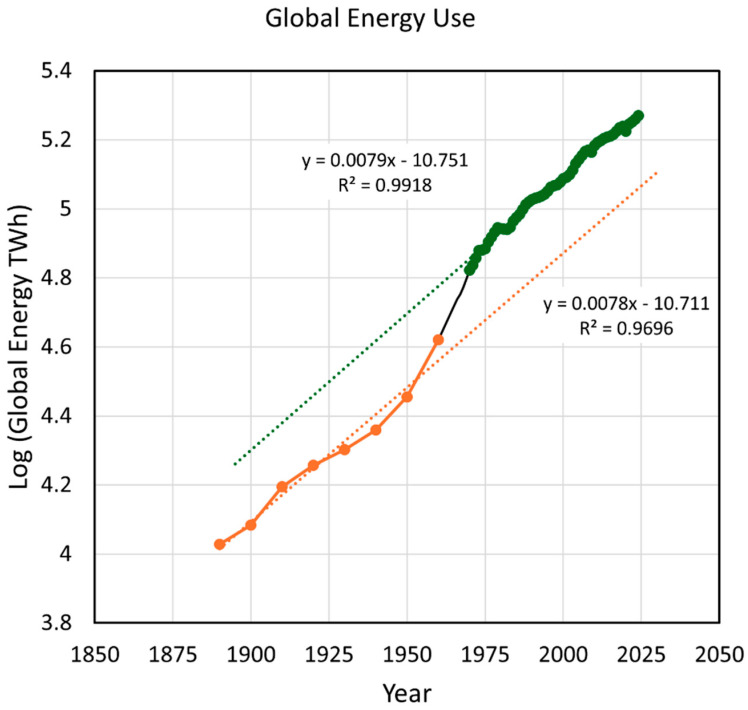
Log-linear plots of global energy use versus year. Regression lines were calculated separately for the green and orange data points.

**Figure A2:**
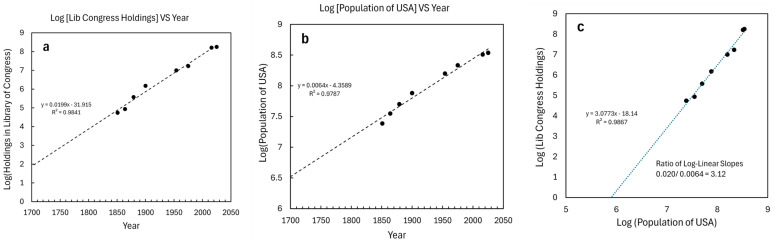
Log-linear plots of Library of Congress holdings and USA populations plus allometry-style plot. See text for details. (**a**) Plot of the log of holdings in the Library of Congress, USA, as a function of year; (**b**) Plot of the log of the USA population versus year; (**c**) Log–log plot of the Library of Congress holdings versus USA population.

**Figure A3:**
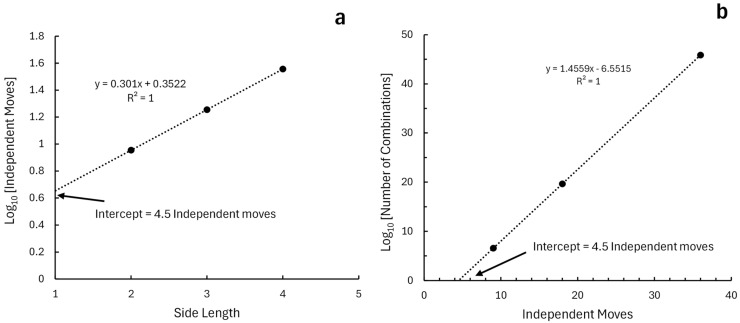
Log-linear plots of complexity components for the Rubik’s cube. See text for details. (**a**) Plot of the log of independent moves versus side length; (**b**) Plot of the log of all possible states (combinations) of the cube versus independent moves.

## Data Availability

All data used in this paper were either previously published by other authors, as detailed in the text, or was obtained from NCBI searches for the taxa mentioned. A Supplemental File has been provided that provides digital versions of data for plots for Figure 1, Figure 2, Figure 3, Figure 4, Figure 5, Figure 6, Figure 7, Figure 10 and Figure 11, and Appendix C. Data values for Figure 8 and Figure 9 were provided in the text.

## Supplementary Materials

The following supporting information can be downloaded at: https://www.mdpi.com/article/10.3390/life16010153/s1.

## Abbreviations

The following abbreviations are used in this manuscript:

Gyr	Giga year
RDTPM	RNA: DNA: triplet code: protein: membrane architecture of life
NCBI	National Library of Medicine—National Center for Biotechnology Information
JCVI-syn3A	Human-constructed minimal cell
nt	DNA nucleotide base pairs
LUCA	Last Universal Common Ancestor
FUCA	First Universal Common Ancestor
PCA	Principal Component Analysis
FLOPS	Floating Point Operations Per Second
AGC	Apollo Guidance Computer

## Appendix A

### Global Energy Use—Transition in Complexity

Energy usage grows with society size and needs. In the context that both population size and population activity (needs) are a form of complexity then the growth in energy use can be considered a form of complexity growth. The data in Figure A1 was obtained from Our World in Data (https://ourworldindata.org/grapher/global-energy-substitution?v=1&csvType=full&useColumnShortNames=false (accessed on 10 May 2025)). The data were provided with pre-processing using the Substitution Method [85] to harmonize it.

When plotted as the log of global energy use from 1890 to 2024, the trends can be divided into two linear portions with identical slopes but separated by a transition time span between 1950 and 1970. This represents a sudden jump in complexity, as predicted by the algebraic perspective of complexity described by Nehaniv [40]. Their prediction is that such jumps would be no more than a 2-fold increase for a single jump. The transition for global energy use is approximately 1.6-fold. The needs of AI processing may lead to another transition soon.

## Appendix B

Under the conjecture that total complexity can be partitioned into orthogonal components (each with a set of features), one method to determine these components is Principal Component Analysis [41].

Consider the parts of a gasoline engine. We assume that we have no a priori knowledge of what the parts do but we are free to take measurements on each part. The goal is to group these parts by function. If we had the same set of measurements on each part, e.g., composition (Fe, Al, Cu, Cr, …, hydrocarbon type and content …, etc.), single piece, multi-component, attachment holes, pivot holes, wire connectors, tube connectors, similarity to other known parts of known function, etc., then we could do a Principal Component Analysis on the collection and we might obtain component groups that we could label in the following way:

(1) Moving components—gears, shafts, pistons, …
(2) Structural components—engine block, brackets, housings, …
(3) Electrical energy components—wires, computer, spark plugs, alternator, …
(4) Chemical energy components—carburetor, gasoline pump, catalytical converter, …
(5) Lubrication components—oil chambers, oil pump, grease chambers, …
(6) Cooling components—fan, radiator, coolant chambers, water pump, …
(7) Energy transfer components—belts, pulleys, transmission, …

As in all Principal Component Analyses, an individual part may appear in more than one principal component with its contributions represented by fractions of 1.0. The larger the fraction, the greater the non-random degrees of freedom the part contributes to the particular component. The linear sum (superposition) of the principal components recreates the complexity of the whole. Since the principal components are orthogonal, each can be examined separately as a partial representation of the total complexity.

## Appendix C

### Library of Congress Holdings and the USA Population

The Library of Congress holdings (a compilation created from various online sources) were plotted versus calendar year in a log-linear scaling. This renders a straight regression line (Figure A2a). Similarly, the USA population (https://www.statista.com/statistics/1067138/population-united-states-historical/?srsltid=AfmBOorME-C01Biak_ggqAOQ6sKgi-4dkWjWhnmK3z2PNZwAXoMXQvyO (accessed on 10 October 25)) was plotted versus year. It also yielded a fairly robust linear regression (Figure A2b). In Figure A2c, the holdings and population are plotted against each other in an allometry-style figure which also has a linear slope. The slope of the allometry-style plot is nearly identical to the ratio of the slopes in panels *a* and *b*. This should be expected, since they both reference the common dimension of time.

Figure A2a might be interpreted as the increase in complexity of knowledge or ideas in the country. Figure A2b might be interpreted as the increase in societal complexity as the number of individuals rises. When plotting these two against each other, one might be tempted to assume they have a causal relationship by suggesting that more books are produced by more people or that more people generate more ideas, etc. It could also be the case that the regressions in panels *a* and *b* are not causally related but simply coincidentally related. When dimension reduction occurs, there is a loss of useful information and inferences may be impaired and should be made with care.

What is useful to extract from the allometry-style plot is the hidden dimension of time. That can be extracted if there are known anchor points to allow interpolation.

## Appendix D

### Rubik’s Cube Example of Hierarchical Complexity

Rubik’s cube is a totally determined puzzle with a defined set of possible combinations of all faces. Three characteristics are shown in the Table. The simplest is the length of a cube side, thereby defining the number of colored panels on each surface of the cube. The second is the number of independent and unique move trajectories that can be made for a given size cube. Finally, the total number of possible combinations of the six colors across all panels of the cube.

To clarify the independent moves, consider a 2 × 2 × 2 cube. One can move the cube bottom (x y plane) a quarter turn, a half turn, or a three-quarter turn. These are independent moves. For the 2 × 2 × 2 cube, rotating the bottom row is the same as rotating the top row in the opposite direction, so these are degenerate. This same set of turns can be deployed on the x z plane and on the y z plane, yielding a total of nine independent moves. It turns out (personal determination) that increasing to the 3 × 3 × 3 cube doubles the number of possible independent moves, and so on for larger cubes.

**Dim**	**Side len**	**Ind Moves**	**Log** **Ind Moves**	**Combinations**	**Log** **Combinations**
2 × 2 × 2	2	9	0.954	3.67 × 10^6^	6.565
3 × 3 × 3	3	18	1.255	4.33 × 10^19^	19.636
4 × 4 × 4	4	36	1.556	7.40 × 10^45^	45.869
1 × 1 × 1	1	Mean(4.5) = 4.5	0.653	1	0

These characteristics define various levels of complexity. Plotting the log of the independent moves versus side length yields a perfect linear relationship (Figure A3a). At the next hierarchical level of complexity, plotting the log of the combinations versus independent moves yields another perfect linear relationship (Figure A3b). These log-linear plots yield examples of the complexity relationship defined in the paper, except that the linear horizontal axis is limited to values of time for biological data.

Each of these regressions can be extrapolated to a 1 × 1 × 1 block predicting 4.5 independent moves. The 1 × 1 × 1 block has no moving parts thus this appears to represent the mean number of independent trajectories one can take when “walking” from face to face around the cube. There are two types of trajectories; one type can successfully land on all five faces (not counting the starting face) and another type ends after four moves because the last face cannot be reached without a jump over a previously landed-on face.
